# High Affinity Antibodies to *Plasmodium falciparum* Merozoite Antigens Are Associated with Protection from Malaria

**DOI:** 10.1371/journal.pone.0032242

**Published:** 2012-02-21

**Authors:** Sreenivasulu B. Reddy, Robin F. Anders, James G. Beeson, Anna Färnert, Fred Kironde, Sharon Kühlman Berenzon, Mats Wahlgren, Sara Linse, Kristina E. M. Persson

**Affiliations:** 1 Department of Microbiology, Tumor and Cell Biology (MTC), Karolinska Institutet, Stockholm, Sweden; 2 Department of Biochemistry, La Trobe University, Melbourne, Victoria, Australia; 3 The Walter and Eliza Hall Institute of Medical Research, Parkville, Victoria, Australia; 4 The Macfarlane Burnet Institute for Medical Research and Public Health, Melbourne, Victoria, Australia; 5 Infectious Diseases Unit, Department of Medicine Solna, Karolinska Institute, Karolinska University Hospital, Stockholm, Sweden; 6 Department of Biochemistry, Makerere University, Kampala, Uganda; 7 Swedish Institute for Infectious Disease Control, Solna, Sweden; 8 Department of Biochemistry and Structural Biology, Lund University, Lund, Sweden; London School of Hygiene and Tropical Medicine, United Kingdom

## Abstract

**Background:**

Malaria kills almost 1 million people every year, but the mechanisms behind protective immunity against the disease are still largely unknown.

**Methodology/Principal Findings:**

In this study, surface plasmon resonance technology was used to evaluate the affinity (measured as k^d^) of naturally acquired antibodies to the *Plasmodium falciparum* antigens MSP2 and AMA1. Antibodies in serum samples from residents in endemic areas bound with higher affinities to AMA1 than to MSP2, and with higher affinities to the 3D7 allele of MSP2-3D7 than to the FC27 allele. The affinities against AMA1 and MSP2-3D7 increased with age, and were usually within similar range as the affinities for the monoclonal antibodies also examined in this study. The finding of MSP2-3D7 type parasites in the blood was associated with a tendency for higher affinity antibodies to both forms of MSP2 and AMA1, but this was significant only when analyzing antibodies against MSP2-FC27, and individuals infected with both allelic forms of MSP2 at the same time showed the highest affinities. Individuals with the highest antibody affinities for MSP2-3D7 at baseline had a prolonged time to clinical malaria during 40 weeks of follow-up, and among individuals who were parasite positive at baseline higher antibody affinities to all antigens were seen in the individuals that did not experience febrile malaria during follow up.

**Conclusions/Significance:**

This study contributes important information for understanding how immunity against malaria arises. The findings suggest that antibody affinity plays an important role in protection against disease, and differs between antigens. In light of this information, antibody affinity measurements would be a key assessment in future evaluation of malaria vaccine formulations.

## Introduction

Malaria is a parasitic infection that threatens almost half of the world's population, with an estimated 243 million cases and around 863,000 deaths in 2008 [1]. Individuals living in malaria-endemic areas who do not die from the disease at a young age, eventually develop immunity against the disease, but only slowly and only after repeated exposure [2]. At a later stage, the capability of controlling parasitemia in the blood is developed. The mechanisms underlying development of anti-disease immunity and the factors governing effective protection are still largely unknown. However, it is well established that antibodies contribute to protection against clinical malaria due to *P. falciparum*. Passive transfer of immunoglobulins from immune donors to individuals with *P. falciparum* infection reduced parasitemia and clinical symptoms [3], [4]. Antibodies directed against cell surface proteins of either the merozoite form of the parasite or of infected red blood cells have been shown to be important components of acquired protective immunity against malaria [5].

In this study, we investigated the protective effect in humans of antibodies against *P. falciparum* merozoite antigens, with a specific goal of looking at whether the affinity of antibodies is of any importance. The Merozoite Surface Protein 2 (MSP2) and Apical Membrane Antigen 1 (AMA1) are well-characterized candidate vaccine antigens and appear to be important immune targets [5], [6], [7]. Therefore, these antigens were chosen to be included in our study. MSP2 is an unusually hydrophilic protein and the monomeric recombinant proteins are largely unstructured in solution but, as a component of the fibrillar surface of *P. falciparum* merozoites, the parasite antigen is probably more structured [8], [9]. This study was focused on the two main alleles of MSP2 (3D7 and FC27), because nearly all *P. falciparum* isolates can be classified into these two major groups. We also wanted to include a protein with a more stable structure, and chose AMA1, which is stabilized by eight intramolecular disulphide bonds [10], [11] and is a type 1 integral membrane protein that is expressed in both sporozoite and merozoite stages of the parasite [12], [13].

Studies on acquired immunity to malaria including merozoite proteins have mostly investigated antibodies to antigens in standard immunoassays using recombinant proteins (eg. ELISA), and there have been inconsistent associations between ELISA results and protection from disease [14]. By eluting bound antibodies with thiocyanate solutions of varying concentrations, ELISA has been adapted to estimate relative antibody affinities [15]. Using this method, trials in both mice and humans have indicated that there are both low- and high affinity antibodies acquired to malaria antigens [16], [17].

The affinity or ‘functional affinity’ [18] of an antibody for its corresponding antigen, has been modeled to be an important determinant of the antibody's biological efficacy [19], [20] and it has been frequently suggested that higher affinity antibodies are more potent than lower affinity antibodies [21]. Also, excessive production of low affinity antibodies has been considered as an expression of immunodeficiency [22], [23] and antibody affinity is believed to be involved in the immunopathology of autoimmune and immune complex diseases [20], [24], [25]. For other pathogens, such as bacteria, it has been shown that affinity of antibodies is important for protection from disease after vaccination [26], [27].

Estimations of the affinity of antibodies in serum has been limited, but with new methods based on surface plasmon resonance, association and dissociation between antigen and antibodies in a continuous flow can be studied in real time. This method has proven to be of great value for evaluation of vaccine design and efficacy studies for other pathogens [28]. In malaria, SPR has mainly been used for evaluation of binding of monoclonal antibodies [29], [30], [31].

The aim of this study was to increase the knowledge about the mechanisms behind the development of antibody-mediated protection against clinical malaria, by investigating whether there are differences in affinity between naturally acquired antibodies directed against different *P. falciparum* antigens, and whether affinity is associated with protection from symptoms of malaria. Affinity of an antibody is usually defined as a constant, KD (dissociation constant) or KA (association constant), for a single binding site at equilibrium. This can be calculated from dissociation- and association rate constants obtained by kinetic measurements. However, association rates are concentration dependent and the concentrations of naturally acquired antibodies in patient sera are difficult to measure, since the antibodies might be directed against hundreds of different epitopes and the concentration of each antibody can be very low. The restricted volumes of the sera also make it difficult to purify antibodies to high concentrations. We have therefore chosen to focus our studies on measurement of dissociation rates (k^d^), as an estimation of affinity since this parameter is concentration independent and can be measured directly in patient sera when using SPR. To have a low dissociation rate is probably a prerequisite for an antibody to be able to exert its function; if it comes off its target antigen too quickly, it will not have time to inhibit for example merozoite invasion. Since the sera can be used without purification, there is no loss of antibodies and we believe that this is important for including all varieties of polyclonal antibodies (both those of high and low affinity) that are present in human sera. Also, since studies on malaria immunity are performed in children and thus only small sera volumes, purification of antibodies is difficult. In this study, we used two representative *P. falciparum* antigens (MSP2 and AMA1) for investigation of affinity (i.e. k^d^) using human samples and monoclonal antibodies. To evaluate the SPR method, we also compared the results with standard ELISA and relative affinities determined by ELISA with thiocyanate elution [32].

## Materials and Methods

### Reagents and genotyping

Surface plasmon resonance (SPR) measurements were performed using a Biacore 3000 instrument, (Pharmacia Biosensor AB, Uppsala) and all reagents including sensor chips CM5, amine coupling kit containing *N*-hydroxysuccinimide [NHS), *N*-ethyl-*N′*-[3-diethylaminopropyl) carbodiimide (EDC), ethanolamine hydrochloride and HBS-EP (10 mM Hepes (pH 7.4), 150 mM NaCl, 3 mM EDTA, 0.005% (v/v) polysorbate 20) running buffer, were from Pharmacia Biosensor AB (Uppsala, Sweden). Sodium acetate, ethylenediaminetetraacetate (EDTA) disodium salt, bovine serum albumin (BSA), NH_4_SCN, and Tween20 were from Sigma.

Genotyping of *msp2* was performed using a nested PCR method [33], to characterize whether parasites contained alleles of the FC27 and/or 3D7/IC type.

### Serum samples

Samples collected from *P. falciparum* exposed individuals in Uganda and in Tanzania were analyzed in this study [34], [35]. The Ugandan samples (n = 48) were collected in Apac in a study from febrile children (6 months-5 years of age) with non-severe malaria (parasitemia from 0.8–10%). The Tanzanian samples (n = 171, age 1–74 years) were collected in a cross-sectional survey in March–April 1999 within a longitudinal population study in Nyamisati, Rufiji district, before the rainy season started. This area has perennial transmission and some seasonal fluctuation. The overall parasite prevalence was 27% and 46% by microscopy and PCR respectively, with the highest prevalence (74% by PCR) observed in children aged 3–5 years [34]. Malaria episodes were continuously recorded through a passive case detection system in which the villagers reported to the unit in the event of fever, for diagnosis of malaria with microscopy, and free treatment. The research unit also served as the only health station in the village. All individuals with fever and *Plasmodium* parasites were treated with sulphadoxine–pyrimethamine. In our study, we included only individuals who were asymptomatic at baseline and had no record of a clinical malaria episode 4 weeks before and 1 week after the survey. All Tanzanian individuals had been tested HIV negative and 77 individuals were *P. falciparum* positive by microscopy at baseline. 55 individuals experienced an episode of malaria during the 40 weeks of follow up (defined as cases), and for every case 2–3 age matched controls were selected among individuals without episodes.

### Ethics statement

Written informed consent was received from all subjects or their guardians, and ethical permissions were granted for the studies from National Institute for Medical Research in Tanzania, Makerere University Faculty of Medicine Research and Ethics Committee in Uganda, and the Stockholm Ethical Review Board (permission numbers 03-095 for the Ugandan samples and 00-084 for the Tanzanian samples).

### *P. falciparum* specific antibody analysis

Determination of anti-*P. falciparum* IgG antibody levels by standard ELISA has been described for the Tanzanian cohort [36]. Microtiter plates were coated with crude parasite antigen solution (*P. falciparum* laboratory line F32, 20 µg/ml), and goat anti-human IgG was used to detect IgG in the plasma samples.

IgG was isolated from sera using Protein G Sepharose 4 Fast Flow (GE health care, Uppsala, Sweden), devoid of the albumin-binding region.

For determination of antibodies against merozoite antigens, ELISA plates (Maxisorb; NUNC 44-2404-21; Denmark) were coated overnight with 1 µg/mL of purified recombinant protein in coating buffer (15 mM Na_2_CO_3_ and 35 mM NaHCO_3_; pH 9.6), blocked with 5% skimmed milk powder in 0.1 M phosphate buffered saline (PBS) pH 7.4, washed ×3 with 0.1% Tween/PBS and then serum samples were added (1∶100 dilution). After another washing step, goat anti-human IgG ALP (Sigma A9544) was applied, *p*-nitrophenyl phosphate tablets (Sigma N2765) used as a substrate and OD measured at 405 nm. All sera were analyzed in triplicate.

The sera were also tested for reactivity against His-tag (Abbiotec, California) in Elisa. Hexa-His was coated on plates at a concentration of 1 µg/well and then Elisa was run as above. The sera showed no reactivity (not shown).

### Proteins and Monoclonal antibodies

Recombinant MSP2-FC27 and MSP2-3D7 proteins with hexa-His tags at the C-termini of the proteins, and the AMA1-D10 protein, with an N-terminal hexa-His tag, were expressed in *E. coli* and purified by metal-chelation on a NTA resin and other chromatography procedures [7], [8]. AMA1-D10 contains the whole ectodomain [37]; the D10 allele of AMA1 is very similar to 3D7 and is identical in domain I. The monoclonal antibodies (mAb) were produced in Australia using standard procedures and spleen cells from mice immunized with recombinant MSP2-3D7 and AMA1-3D7. The MSP2 mAb 6D8 recognizes the conserved N-terminal region. MAbs 9G8, 4D11, 6C9, 1F7, and 9H4 are directed against epitopes in the conserved C-terminal region, and mAbs 11E1, 2F2, and 9D11 recognize epitopes in the 3D7 family specific region (Table 1). MAb 8G10, which recognizes an epitope in the 32-mer repeat of MSP2-FC27, was provided by the Queensland Institute of Medical Research. Three anti-AMA1 mAbs were included in the study; 1F9 recognizes a polymorphic conformational epitope at one end of the solvent-exposed hydrophobic trough on the AMA1 ectodomain [10], mAb 2C5 binds to the correctly folded ectodomain but the epitope has not been mapped, and mAb 5G8 recognizes a linear epitope N- terminal to the first conserved cysteine of the molecule [38]. All monoclonal antibodies were diluted in HBS-EP running buffer.

**Table 1:** Binding sites for MSP2 monoclonal antibodies.

	MSP2-Fc27	MSP2-3D7
**6D8**	N-term const	N-term const
**9G8**	C-term const	C-term const
**4D11**	C-term const	C-term const
**6C9**	C-term const	C-term const
**1F7**	C-term const	C-term const
**9H4**	C-term const	C-term const
**8G10**	Fc27 variable	-
**11E1**	-	3D7 variable
**2F2**	-	3D7 variable
**9D11**	-	3D7 variable

Indication of binding sites for the MSP2 specific monoclonal antibodies. Const = directed against constant regions of MSP2, variable = directed against variable regions.

### Surface Plasmon Resonance

CM5 sensor chips were used together with N-terminal amine coupling kits from Biacore to bind the proteins to the chip. The carboxymethylated dextran surface of the chip was activated using an injection pulse (10 min, 5 µL/min) containing a mixture of 0.05 M NHS and 0.05 M EDC. The protein was immobilized by manual injection at 100 µg/mL in coating buffer (0.01 M sodium acetate buffer, pH 4.0) until the desired response units were achieved. Before coupling, an SDS-PAGE was run to confirm that each protein consisted of a single band (data not shown). The remaining sites on the sensor surface were blocked by injecting 50 µL of 1 M ethanolamine (pH 8.5) for 10 minutes. All steps were carried out in a continuous flow of HBS-EP running buffer at 5 µL/min, and all buffers were degassed prior to use.

The SPR binding assays were performed with a constant flow rate of 30 µL/min at 25°C. Serum samples (in different dilutions, 1∶7.5, 1∶15, 1∶30, 1∶60 or 1∶90) or purified mAbs were flowed over the bound recombinant proteins in HBS-EP buffer, pH 7.4. At all dilutions, the association of antibodies with the immobilized proteins was monitored for 3 minutes followed by 10 minutes of dissociation. Residual bound antibody was removed by washing the chip with 10 mM glycine-HCl (pH 1.5) for 5 seconds at 5 µL/min. Reequilibration between the sensor surfaces and running buffer was established prior to injection of the next sample. Response was monitored as a function of time (sensogram) at 25°C. All SPR measurements were performed in at least two different dilutions of sera. Kinetic parameters (k^a^ and k^d^) were evaluated by fitting the data using the BIAevaluation 4.1 software.

### Ammonium thiocyanate ELISA

ELISA plates (as above) were coated with purified recombinant protein (1 µg/mL) in coating buffer, blocked with 1% BSA/PBS, washed ×3 with 0.1% Tween/PBS and then serum samples (1∶100) were added followed by washing ×3. NH_4_SCN was added in different dilutions (0.0, 0.25, 0.5, 1.0, 2.0 and 3.5 M) and incubated for 15 minutes at room temperature. After this, the procedure was the same as above for the ELISA.

### Statistical analysis

Statistical analyses were performed with GraphPad Prism 5.0 software and R 2.13.0. To test for differences in mean affinity between the recombinant proteins, an analysis of variance was performed on the natural logarithm of the original measurements. If there was evidence to reject the null hypothesis (no difference in means), the Tukey Honestly Significant Differences test was used for pair wise tests. For the Tanzanian samples, a binomial regression was conducted to test whether a high affinity was associated with the probability of getting malaria during follow up. As independent variables we included a dichotomic variable whether a malaria episode occurred within 40 weeks of follow up, the affinities of each of the three recombinant proteins, and age. To test if affinity was dependent on whether malaria occurred in the years before the bleed, a normal linear regression was fitted to the logarithm transformation of affinity using the protein type, the mean number of malaria episodes per year, total number of episodes before the bleed, and age as covariates. Segmented regression based on a normal linear model was fitted to the natural logarithmic transformation of the affinity to estimate the break-point in age, that is, to determine whether the relationship between age and affinity changed after a certain age. Where it was not possible to determine a break-point, we fitted a normal linear regression without break-points. The analysis was carried out separately for each recombinant protein. Correlations were calculated and tested as Spearman rank correlations. For all tests two-tailed P values were considered significant if they were <0.05, and where models were fitted, residual plots were used to determine the correctness of the model. To analyze the importance of the presence of MSP2-3D7 or MSP2-FC27 parasite genotypes in the blood, a normal linear regression was fitted to the logarithmic transformation of the affinity. As covariates, we included age, protein type, and two dichotomous variables, one indicating the presence of parasites of the MSP2-3D7 type and the other the presence of parasites of the MSP2-FC27 type. An interaction of these two dichotomous variables was also added to indicate whether none or both were found. Residual plots were used to confirm the fulfillment of the conditions necessary for the model.

## Results

### Contrasting affinities of antibodies to AMA1 and MSP2

**Figure 1:**
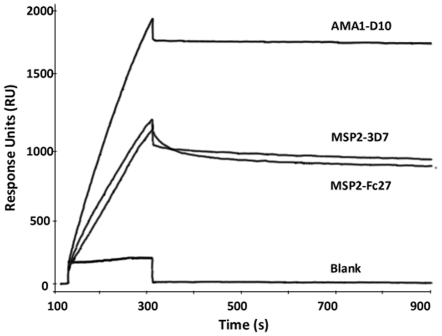
SPR Sensogram for one representative plasma sample. A sensogram showing association and dissociation of a representative serum sample to recombinant MSP2-FC27, MSP2-3D7 and AMA1-D10 (Fc2, Fc3 and Fc4, respectively). Fc1 was used as blank to control for non-specific binding.

Three recombinant merozoite antigens (MSP2-FC27, MSP2-3D7 and AMA-D10) were immobilized through amine coupling to CM5 surface plasmon resonance (SPR) sensor chips. The affinity of antibodies in patient sera for these antigens was estimated using dissociation rates (k^d^) as described above, including a series of different dilutions of the serum samples. We used Swedish non-immune sera to determine the cut-off to be considered as background level ( = 100 RU), and only samples that generated binding data that were clearly discernable above background noise were included in the subsequent analysis. The k^d^ values were independent of the amounts of protein immobilized, demonstrating that the binding constants are independent of protein surface density for the chosen concentrations. Very little or no binding was detected for serum samples injected over the reference surface (Figure 1). The dissociation rates were also independent of the serum concentration used. In order to test the stability of the recombinant proteins on the chip surface, a pool of sera (from immune individuals) was used as an internal control after every 20 samples. The k^d^ values were independent of the loss of protein over time (which was seen as a decrease in response units from each surface). As a control, anti-IgG was added directly after the serum samples for a subset of ten samples, to confirm the presence of bound IgG to the recombinant proteins.

**Figure 2:**
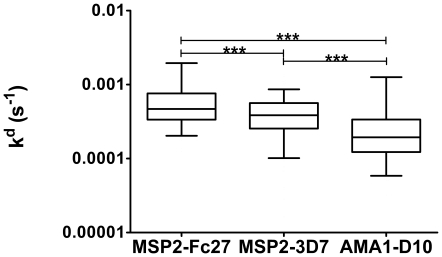
Affinity measured as k^d^ in 219 individuals. Pattern of affinity response (measured as k^d^ values) of exposed sera (n = 219) against recombinant proteins coupled to an SPR-chip. The increasing affinity (equals lower kd values) was observed in the order of MSP2-FC27, MSP2-3D7 and AMA1-D10 and the differences were statistically significant (p<0.0001), both when the individual proteins were compared to each other and compared across all three antigens (ns = p>0.05; * = p<0.05; ** = p<0.01; *** = p<0.001). The box plot values represent the 25^th^ percentile, median, and the 75^th^ percentile. The whisker range is between the 5th and 95th percentiles.

Human serum samples studied were obtained from two field sites, one in Tanzania and the other in Uganda. The Tanzanian samples came from 171 at baseline asymptomatic individuals participating in a cross-sectional survey in a longitudinally followed population, and the Ugandan samples from 48 children with acute uncomplicated malaria. The Ugandan samples were collected in an area with perennial holoendemic presence of malaria with a very high transmission rate [39]. With these two sets of samples, we could compare affinities of antibodies in different areas, as well as different age groups and levels of disease and endemicity. When a total of 219 samples were investigated, significant differences (p-values<0.001) in affinity between the proteins were observed, with antibodies to AMA1 having higher affinity than antibodies to MSP2, as shown in Figure 2. In addition antibodies to MSP2-3D7 had higher affinities than antibodies to MSP2-FC27. The results were the same when each population (Uganda or Tanzania) was analyzed separately and the same pattern was seen for all age groups. The differences were significant both when the antigens were individually compared to each other, and when the comparisons were made across all three antigens. Out of 219 samples tested, 90% had measurable responses in SPR for MSP2-3D7, 83% for MSP2-FC27 and 90% for AMA1-D10. No common factors (such as age) could be found for the samples that did not have measurable responses. The samples that had enough response to be classified as significant binding usually reached levels of Response Units of several hundreds-to-thousands within the three minutes used for our experiments (see, for example, Figure 1), indicating that the association rates were relatively fast for all samples. To establish that the effects of whole serum binding to recombinant proteins on the SPR chip was predominantly due to antibodies, IgG was purified from 5 of the Tanzanian samples (from individuals aged 12–51 years, 3 *P. falciparum* positive and 2 *P. falciparum* negative by microcopy at baseline) and tested against MSP2-3D7 and MSP2-FC27. There were no significant differences between the k^d^ values obtained with purified IgG samples and the corresponding whole sera (data not shown), indicating that the majority of the measured effect was due to antibodies present in serum that specifically bound to the immobilized antigens.

**Figure 3:**
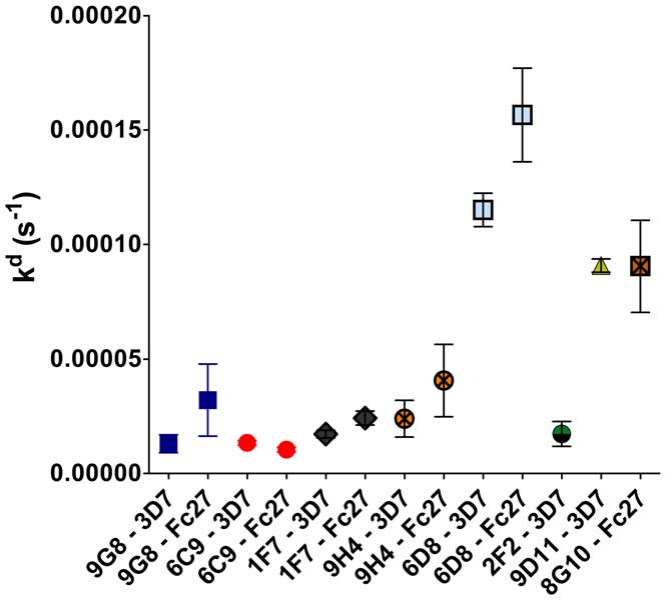
kd values for monoclonal antibodies against MSP2. Comparison of affinity response of the mAbs against recombinant MSP2-FC27 and MSP2-3D7 proteins. The error bars indicate mean ± SEM.

The affinities of antibodies in serum samples were compared to the affinities of sets of mAbs directed against AMA1 and MSP2. Initially, we analyzed the affinities of mAbs 1F9, 2C5 and 5G8 when binding to AMA1-D10 on the SPR-chip. 1F9 was found to bind with very high affinity (k^d^ = 6.2×10^−6^), while 2C5 and 5G8 showed lower affinities (k^d^ = 4.2×10^−4^ for 2C5, and k^d^ = 9.2×10^−4^ for 5G8). We also analyzed several mAbs against MSP2 (Table 1, Figure 3), and found that when the k^d^ values for the mAbs against both AMA1 and MSP2 were compared with those found in patient sera, they were similar, except for 1F9 which showed a very high affinity. For the MSP2 proteins, the mAbs 9G8, 1F7 and 9H4 (directed against the conserved C-terminal region) exhibited slightly higher affinity for the 3D7 allele compared to the FC27 allele, as did also mAb 6D8 directed against the N-terminal conserved region although this mAb had a lower affinity in general. The mAbs 9D11 and 8G10, directed against the variable regions of MSP2-3D7 and MSP2-FC27, respectively, had relatively low affinities while mAb 2F2, directed against the variable region of MSP2-3D7 had a higher affinity. The affinity of 4D11, directed against the conserved C-terminal region, was too low to measure, as was also mAb 11E1, directed against the variable region of MSP2-3D7.

To evaluate the effect of different mixtures of antibodies, ten different serum samples (selected because we had relatively large volumes of these) were analyzed using SPR and the MSP2-FC27 protein. The individual k^d^ values ranged from 3.3×10^−5^ to 7.8×10^−4^ s^−1^. The theoretically calculated mean for these samples was 2.9×10^−4^, (median 2.3×10^−4^) but when an equal mixture of all ten samples was studied, the measured k^d^ value was 1.6×10^−4^ s^−1^. This indicates that when antibodies are mixed, such as for example in serum, the values measured are representatives of the mixtures of antibodies, and not only a representation of one dominating high-affinity antibody. When an analogous experiment was performed with mixtures of mAbs, the results were similar. For example, the individual k^d^ values for the mAb 9G8 and 6D8 against MSP2-3D7 were 1.30×10^−5^ and 1.15×10^−4^ s^−1^ respectively, whereas the 50∶50 mixture of these antibodies produced a k^d^ value of 5.73×10^−5^ s^−1^. The mAb 6C9 had an individual k^d^ value for binding to MSP2-3D7 of 1.34×10^−5^, while a mixture of 25% 6C9 and 75% 6D8 gave a k^d^ value of 7.12×10^−5^ s^−1^, and 75% 6C9 and 25% 6D8 gave 1.35×10^−5^ s^−1^.

### Correlation between affinity and ELISA results

To compare the affinity of the antibodies in serum samples to total IgG/IgM as estimated by ELISA, we used the recombinant proteins for both SPR with a continuous flow of sera containing the antibodies, and standard ELISAs, for a subset of samples. Initially, the 48 sera collected from children in Uganda (6 months–5 years of age) with mild malaria were used. These samples were selected for comparative studies, since we had relatively high volumes of these samples. 44 sera gave measurable responses for all antigens in SPR and were therefore used also for ELISA studies. All the 44 samples were also positive in ELISA assays for all antigens. No significant correlation was seen between standard IgG ELISA and SPR results for the MSP2 proteins (Spearman correlation coefficient −0.22 for MSP2FC27 and −0.25 for MSP23D7), however the IgG ELISA values correlated with SPR for the AMA1-D10 (Spearman correlation coefficient −0.37, p = 0.01). When standard IgM ELISA was compared to SPR assays for all three proteins, there were no significant correlations.

The samples from Uganda were all from symptomatic children with *P. falciparum* malaria. When the comparison between IgG schizont extract ELISA [34] and SPR data was performed on the Tanzanian cohort where all the samples were from asymptomatic individuals, we found no correlation between *P falciparum* specific IgG and our SPR data generated with the recombinant proteins.

### Effect of age and concurrent infection on antibody affinity and ELISA results

**Figure 4:**
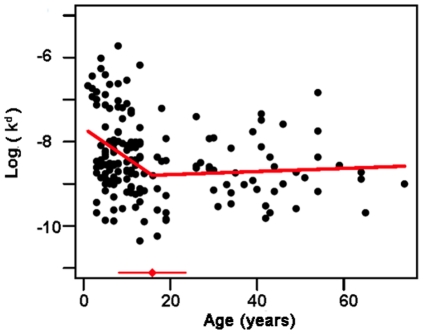
Natural logarithm of k^d^ variation with age in the Tanzanian population. Affinity variation with age for samples from Tanzania, shown for binding to AMA1-D10 where the break-point was determined to be 16 years of age. The solid line is the estimated fit from the linear normal model. The red dot with a line at the bottom indicates the 95% confidence interval for the breakpoint.

Since protection against malaria is often associated with older age in endemic areas, we wanted to know how affinity of the antibodies developed with age. A segmented regression analysis was used for the sera from Tanzanian individuals, where we had access to a relatively large number of samples (n = 171) of different ages (1–74 years) to estimate a possible break-point for age in terms of affinity. For AMA1, the affinity increased until 16 years of age where there was a breakpoint after which affinity values were stable (p<0.01, Figure 4). For MSP2-3D7 no breakpoint could be determined, but a slight increase in affinity with age could be seen when all ages were considered together (p<0.05). For MSP2-FC27, no significant changes in affinity with age could be seen.

For *P. falciparum* (prepared as schizont extract) specific IgG there was a significant increase with age (p<0.0001) in the Tanzanian sera [34]. For the samples from symptomatic children in Uganda (aged 6 months–5 years), no significant correlations between age and IgG ELISA could be seen for the MSP2 proteins (each protein was analyzed separately), but for AMA1-D10 there was a significant correlation between IgG and age (p<0.0001, Spearman correlation coefficient 0.64).

### Ab affinity and protection from malaria

**Figure 5:**
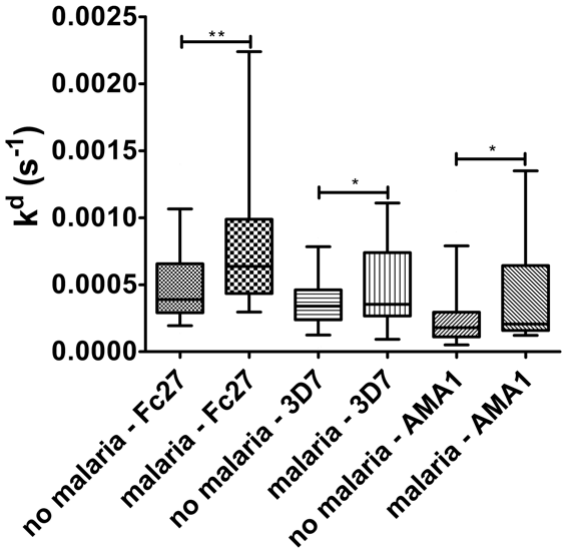
Association between affinity (measured as k^d^) and protection from clinical malaria. Affinity versus protection for the individuals in the Tanzanian population that were *P. falciparum* positive (n = 77). Individuals with high affinity had lower risk of developing malaria during follow-up (No malaria = did not get malaria within 40 weeks, Malaria = Experienced a clinical episode of malaria during follow up). Fc27 and 3D7 indicates the recombinant MSP2 proteins used in the SPR assay. (Box plots representations as in Figure 2.)

The Tanzanian samples were from asymptomatic individuals of all ages who were followed prospectively with regards to febrile episodes and malaria. These samples were therefore used for further analysis of associations between affinity, IgG ELISA results and protection from malaria. When the time to the next clinical episode of malaria from baseline was assessed it was found that the 17 individuals having the top 10% of the highest affinity antibodies (out of 171 samples) against MSP2-3D7 (mean kd 1.10×10^−4^) had a longer duration (mean 37 weeks) to clinical malaria when compared with individuals with the 10% lowest affinity antibodies (mean kd 9.10×10^−4^, duration 25 weeks, p<0.0001). High affinity against MSP2-FC27 or AMA1-D10 did not correlate with longer time to disease. We also analyzed whether there was any association between how many times the individuals had malaria in the years before the survey and affinity for the different antigens, but could not find any associations (either as episodes per year or as total number of episodes). The individuals were then divided into two groups: those that had parasites in the blood at the time of sampling, and those that were parasite negative, similar to approaches used in other studies [40] because parasitemia has been associated with antibody levels. Further, the samples were subdivided into those in whom malaria was diagnosed within 40 weeks of follow up, and those who did not develop malaria. There was no significant difference in *P. falciparum* specific IgG between malaria prone and protected individuals (whether analyzed as parasite positive and negative separately, or all samples together, and including adjustment for age). In contrast, among individuals who were parasite positive at baseline (n = 77), higher antibody affinities to all antigens were seen in the individuals who not experience febrile malaria during follow up (Figure 5, p<0.01 for MSP2-FC27, p<0.05 for MSP2-3D7 and p<0.03 for AMA1-D10), suggesting that high affinity may be important for protection against disease. In the parasite negative group, no differences in affinity could be seen between malaria prone and protected individuals.

### The importance of allelic variant present in the blood

**Figure 6:**
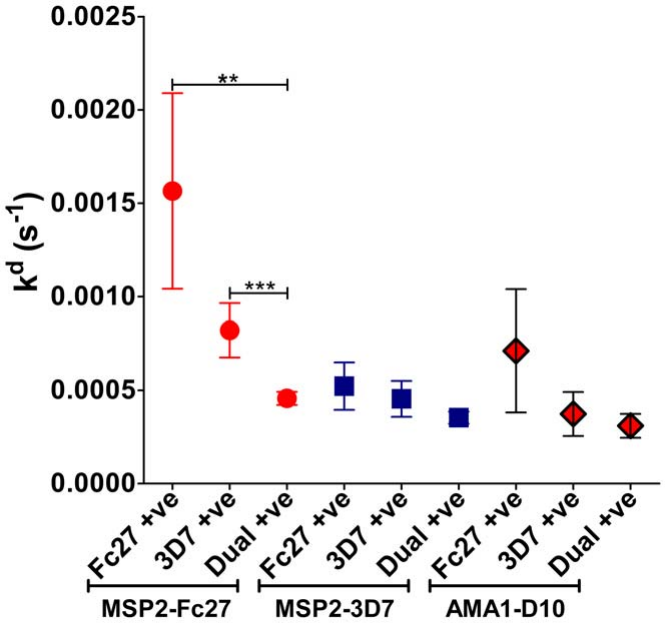
Presence of specific alleles of parasites in the blood associated with different affinities. Affinity of Tanzanian serum samples depending on which allele of MSP2 parasite was present in the blood at the time of collection. Presence of both alleles of MSP2 is associated with the highest affinity of antibodies in the sera, and that presence of the 3D7 allele was associated with higher affinity compared to the Fc27 allele. The error bars indicate mean ± SEM, and the asterisks indicate the level of significant differences (ns = p>0.05; * = p<0.05; ** = p<0.01; *** = p<0.001).

We investigated whether it made a difference in affinity which *P. falciparum* MSP2 genotypes were present in the blood of the individuals at the time of sample collection in the Tanzanian cohort (Figure 6). 39 individuals were infected with parasites of both the 3D7 and FC27 MSP2 alleles, 28 had only the 3D7 allele and 10 had only the FC27 allele. When MSP2-FC27 was the target protein in the SPR analysis higher antibody affinities were seen in individuals infected with parasites having the 3D7 MSP2 allele (p<0.03), and with parasites of both MSP2 genotypes in the blood at the time of sample collection (p<0.005). These results reached significance only when antibodies against MSP2-FC27 were used in the SPR analysis, and the results obtained using MSP2-3D7 or AMA1-D10 as target proteins showed a comparable trend. Similar observations were made with the Ugandan samples in which presence of 3D7 allele was associated with higher affinity for all antigens, but only reached significance using MSP2-FC27 as the target.

### Comparison of methods

Since SPR analysis is a relatively new method for investigating affinity of antibodies in serum, we performed a comparative analysis of SPR and ammonium thiocyanate ELISA for the samples from Ugandan children (these samples were used since we had relatively large volumes of them) using the recombinant MSP2-FC27 and 3D7 proteins. The ammonium thiocyanate ELISA was performed as an ELISA (measuring IgG) but with addition of increasing concentrations of ammonium thiocyanate. No correlation was seen between SPR and ammonium thiocyanate ELISA, but a correlation was observed between standard IgG ELISA and ammonium thiocyanate ELISA for both MSP2-FC27 and MSP2-3D7 (p<0.0001 for both, Spearman correlation coefficient 0.66 and 0.70, respectively).

## Discussion

We have investigated affinity (measured as k^d^) of naturally acquired antibodies in serum against two important immune targets and leading *P. falciparum* blood-stage vaccine candidates, AMA1 and MSP2. We found that high affinity against either of the antigens (although significant only among individuals who were parasite positive at baseline) was associated with protection from experiencing disease during follow up in a high endemic area in Tanzania. Moreover, the individuals with the highest affinities against MSP2-3D7 had longer time to next episode of malaria. Affinity increased with age for AMA1 and MSP2-3D7. This indicates that affinity is important for development of protection against malaria, and also that affinity needs to be taken into consideration when evaluating new vaccine candidates. A protective effect of affinity could not be seen in individuals who were parasite negative at baseline; this might be because our study population was too small to make the differences significant, or because the exposure to parasites in general was lower in the parasite negative group.

When *P. falciparum* IgG was measured in a standard ELISA, it was not associated with protection, even though the levels of antibodies increased with age. This supports the idea that function and not only presence of antibodies is important for protection against malaria. It might be that affinity increases with increased exposure to malaria, but since there were such clear differences between different antigens and the results were adjusted for age, and because there was very little correlation between ELISA results (which are often considered to be associated with exposure [41]) and affinity, we think that an evaluation of protection against malaria should include a consideration of antibody affinity.

In both populations studied antibodies to AMA1 were of higher affinity than antibodies to MSP2. In the area in Uganda, transmission rates are extremely high and the samples were from febrile children. In Tanzania, the area has some seasonal fluctuations in malaria transmission and the samples were from individuals without symptoms. In spite of these dissimilarities, the affinity patterns towards AMA1 and MSP2 proteins were the same. The differences in affinities could be related to different structural characteristics of these two antigens. AMA1, which is conserved throughout the *Plasmodium* genus and in other apicomplexan parasites, has globular domains stabilized by intramolecular bonds [11], [42]. In contrast, MSP2, which lacks orthologues in most *Plasmodium* species, is an intrinsically unstructured protein [8], [43], which could potentially result in relatively low affinity antibodies [44]. High affinity antibodies result from the selection by antigen of relatively rare B cells in which the process of somatic hypermutation (with the help of follicular T helper cells) generates B-cell receptors that bind antigen with higher affinity. The increased affinity of the matured antibody response for conventional antigens reflects an improved fit between antigen and antibody so that affinity is not limited by the entropic cost of conformational changes in the antibody complementarity determining regions, which are a characteristic of the induced fit interactions of antibodies in the naïve repertoire [45], [46]. When unstructured proteins bind target ligands, they usually adopt a more ordered conformation [47] and we assume that MSP2 would undergo similar transitions when binding to many antibodies. The entropic cost of these transitions could limit the affinity of MSP2-antibody interactions irrespective of how effectively the process of somatic hypermutation generated high affinity anti-MSP2 antibodies.

The different alleles of MSP2 can be categorized into two major groups, 3D7 and FC27, based on differences in repeats and flanking variable sequences [48], [49], [50]. Parasites of both allelic types have been shown to be present in Tanzanian and Ugandan populations, although the 3D7 allele appears to be slightly more common [34], [51], [52], [53]. It was noted that both human polyclonal antibodies and several of the mAbs directed against conserved regions of MSP2 bound with higher affinity to MSP2-3D7 than to MSP2-FC27. It might be that the FC27 form of MSP2 has a lower propensity to adopt the more structured conformation required for some of the antibodies to bind, but it could also be that higher exposure to the 3D7 allele has resulted in formation of higher affinity antibodies.

We noted that the presence of parasites of the 3D7 type in the blood correlated with higher affinity of all antibodies (even though it reached significance only for antibodies to MSP2-FC27), and that high affinity towards the 3D7 allele correlated with longer time to next infection, further strengthening the importance of the 3D7 allele. This is very important knowledge for future vaccine studies, showing that one of the most critical parameters for the outcome of the study may be which allelic form of a protein the vaccine is based on. These results are in line with older studies where strain-specificity for MSP2 was noted to be of importance for formation of a protective response (even though the protection was very modest) [54], and where a higher prevalence of 3D7-specific antibodies was found in children with asymptomatic malaria and a higher prevalence of FC27-specific antibodies was found in children with clinical malaria [55]. Our findings are also important for understanding how development of protection against malaria arises; if particular allelic forms of antigens generate higher affinity antibodies, the rate at which protection against disease develops may vary depending on which parasite genotypes are encountered.

For AMA1, several polymorphisms have been described. In our studies, only the D10 form of AMA1 was used and different results may have been obtained if another form of this antigen had been used. However, there are descriptions in the literature of antibodies that bind to several variants of AMA1 [56], and even one monoclonal that binds to an epitope conserved on all strains of *P. falciparum* examined [29]. We could conclude that most of our tested sera were positive for antibodies against AMA1, indicating that either the used strain is common in the population, or that it can cause development of cross-reactive antibodies against AMA1. It is difficult to speculate what the result would have been if other strains had been used, but our results show that there is at least one strain that can generate high-affinity antibodies in patients, which strengthens the potential use of AMA1 as a vaccine candidate.

We have used the dissociation rate constant, k^d^, as an indicator of affinity, because determination of k^d^ is not reliant on knowledge of the concentration of the antibodies, which is difficult to assess in polyclonal samples [26]. Judging from the levels of Response Units achieved within just a few minutes of binding between antibody and antigen, the association phase of binding was in general very effective, indicating that the antibodies may be functional in for example binding to merozoites, which are only accessible to antibodies for a short time. It has been suggested that antibodies would need to exist at high concentration to be effective in inhibiting merozoite invasion due to this kinetic constraint and antibody affinity would be of less importance [57]. However, the importance of affinity for activity in inhibiting merozoite invasion was shown in a study with anti-AMA1 shark antibodies (IgNARs) [58]. In this study mutagenesis of an IgNAR resulting in an increase in the affinity for AMA1 (a decreased dissociation rate but little change in the association rate) was associated with an increase in the merozoite invasion inhibitory activity. Also, in vivo, binding of one antibody might slow down the invasion process, allowing more time for other antibodies to bind, causing relatively low concentrations of several antibodies to become functionally important. In future studies, we intend to measure dissociation rates against antigens on the infected erythrocyte surface, to establish whether high affinity is something that is developed in an individual at a certain stage of immunity, or whether it is different and specific for each antigen that an individual is exposed to.

We could show that the affinity range (measured as k^d^) in the sera was similar to that for monoclonal antibodies, with the exception that mAb 1F9 bound to AMA1-D10 with very high affinity. mAb 1F9 has previously been shown to have a very large binding surface area [38], which no doubt helps in creating a stable complex. 1F9 is known to inhibit invasion, but 2C5 and 5G8 (which are also directed against AMA1) do not. This could be because 1F9 binds a neutralizing epitope, but as 2C5 and 5G8 bind AMA1 with about a hundred times lower affinity than 1F9, affinity may be important for antibody function in the growth inhibition assay, as has also been shown in studies with AMA1-binding shark antibodies [58]. It has been shown before that function and not only presence of antibodies is important in malaria [59]. In further studies, we will compare affinity to invasion inhibition and see whether these relate to each other.

Our results from the SPR studies are the total result of measurement of a polyclonal response, and we could show that our measurements are the sum of binding of many different antibodies by mixing different sera and different mAb. There might have been a low concentration of very high affinity antibodies in the sample that we could not detect, but if there at the same time was a high concentration of lower affinity antibodies, the net effect would probably still be that the binding was weaker with antibodies coming off the target protein more quickly. It has been shown before that mixing of different antibodies can affect the total outcome of the function of the antibody [60].

In this study, we also wanted to evaluate different methods for measurement of antibodies. ELISA measures the presence or absence of antibodies in a static system, while SPR measures the strength of the interaction during flow. For AMA1, which bound antibodies with high affinity, we could see a correlation between standard IgG ELISA and SPR methods. This might be because high affinity antibodies are not washed away during handling of the ELISA plate. This would be in line with the fact that we could see a correlation between the MSP2 proteins binding in standard ELISA and in NH_4_SCN ELISA, as these methods are much more similar to each other than to SPR. When NH_4_SCN ELISA assays have been used in malaria research before, either for evaluation of affinities after vaccination or after natural infection, the results have often failed to show consistent patterns [61], [62], indicating that other means of measuring affinity is needed.

In conclusion, we have demonstrated a new way of evaluating antibodies in malaria, using SPR for measurement of affinity in polyclonal responses against different proteins. We have shown that high affinity correlated with longer time to infection during follow-up for MSP2-3D7. Further, the presence in the blood of parasites expressing particular alleles of the antigen was associated with higher affinity antibodies. This information is important for understanding how immunity against malaria arises, and for selection of future vaccine candidates.
